# Bibliographical Notices

**Published:** 1848-03

**Authors:** 


### BIBLIOGRAPHICAL NOTICES.

*Materia Medica and Therapeutics.* By Martyn Paine, A. M., M. D., Professor of the Institutes of Medicine and Materia Medica in the University of New York, etc. New-York: Samuel S. and William Wood, No. 261, Pearl street. 1848. 16mo. pp., 411.

This work is designed as a compendium of Materia Medica and Therapeutics, to conform to the arrangement pursued by the author in his work on the Institutes of Medicine. The several articles of the Materia Medica of each denomination are arranged in the order of their estimated therapeutical value. The pharmaceutical statements usually introduced into works on Materia Medica, and for the most part, physical and botanical characters are ommitted. Remarks on the therapeutical action, &c., of different remedies, are only occasionally introduced, and, when introduced, are very brief. The main object of the work, in short, is to present an enumeration of the various articles of the Materia Medica, grouped after a method of classification, based upon therapeutical principles as taught by the author.

Prof. Paine is one of the very few teachers and writers of the present day, who tenaciously adhere to the doctrines of ultra vitalism. He recognizes among the phenomena of the living organism those forces only which are exclusively vital in their character, and which are inherent in the solid structures. He belongs, therefore, to the school of solidism, as well as vitalism. His views of the therapeutical action of remedial agents, necessarily, differ from those generally entertained on this subject, and we may add, from the dcctrines which are most consistent with the present stage of the progress of Physiology and Pathology. It seems to us these views are so readily and completely refuted by well known facts, that it would be deemed a work of supererogation to undertake their disproval. We hazard the assertion that few, even of those who listen to Dr. Payne's oral instructions, will adopt the opinions inculcated in the following quotation from the work under notice:—

"Remedial agents operate directly upon the vital properties of the parts to which they are applied, and through the medium of those parts, upon remote organs by the principle of sympathy. The partial absorption of certain remedies is only a contingent result, and has little or no agency in the physiological phenomena. Their reputed absorption is greatly overrated, often only imaginary, and sometimes misrepresented."

The charge of misrepresentation we presume is intended to cover those facts which cannot be explained on the plea of exaggeration, or of too vivid an imagination. It is a very convenient alternative when one is driven to a *dernier resort.* This method of argumentation reduced to a methodical shape would stand thus:—(a) the reputed phenomena are overrated; (b) they are imaginary; (c) they are manufactured for the occasion. An opponent with these several strings to his bow, can hardly be vanquished. We are reminded of the argument of the lawyer rebutting the allegation that his client had injured a neighbor's brass kettle: the gentleman of the green bag contended, first, that the kettle was whole when returned:

second, that the hole was in it when borrowed, and, third, that his client never borrowed the kettle! Having proved all these points, he considered that his case was clearly established. In like manner if the reputed absorption of medicinal agents can be proved to be overrated, imaginary, and misrepresented, solidism and vitalism are save from the innovations of the humoral pathology!

Assuming, as the author does in the quotation already given, that medicines act, exclusively upon the vital properties of solid parts, and upon remote parts solely by sympathy, we were curious to see what he would say under the head of *alteratives*, a term which he adopts as the title of an extensive group of medical agents. We quote his preliminary remarks under this head:—

"I have included under the denomination of alteratives all such remedies as, in certain doses and at certain intervals of exhibition, interrupt the progress of disease without any prominent demonstration. The changes which are thus induced are generally gradual, and may or may not be, sooner or later, attended by some evacuation, as with the group of cathartics. Thus, a profuse discharge of saliva may be established by minute doses of calomel, or an equally profuse secretion of bile and consequent diarrhœa; and antimony and ipecacuanha may occasion, in their small alterative doses, copious perspiration. (See *Preface* and *Institutes*, article *Alteratives* in Index.)"

Mercury, given as an alterative, in "dose of from gr. ⅛ to ii. once in two to twelve, or more hours," ranks first in the group; and, according to the author's doctrine, must exert its remedial influences solely by its action on the vital properties of the stomach, affecting the mouth, and other parts of the body, by means of the sympathetical relations of the stomach!

One class in the authors classification is entitled *Chemical Agents* Turning to the section which treats of these, we find their scope defined as follows:—

"Employed to neutralize offending acids, and other substances, in the alimentary canal, and sometimes to operate subsequently, and partly in virtue of such new combinations as cathartics."

We naturally were led to turn from this portion of the book to Genito-urinary agents, in order to ascertain if the author recognized the fact that alkalies taken into the stomach are capable of neutralizing acidity of the urinary secretion, and to see if he would undertake to explain this fact by the principle of sympathy. The ingenuity with which the author escapes from this dilemma is only equalled by the mode in which he disposes of the facts which go to sustain, generally, the reputed absorption of medicinal agents. He says: "the agents embraced in this group possess specific relations to some one or more parts of the urinary and genital organs." He says nothing respecting relations to the urinary secretion, and does not include in this group any of the alkalis, or diuretic salts! We are left to presume that the imputed absorption of substances affecting the chemical constitution of the urine is one of the instances in which the medical world are deluded by overrated, imaginary statements, and by misrepresentations! We here take leave of the work. We are sorry not to be able to bestow upon it our humble meed of praise; but candor compels us to say, that, in so far as it may possibly extend the therapeutical principles to which we have alluded, it will do harm rather than good. Although it may seem like presumption, we cannot forbear to express regret that the learning, talent and zeal of the author should be devoted to the worse than profitless effort to sustain doctrines which have been abundantly disproved by demonstrative evidence, and which are well nigh obsolete.

---

*Ophthalmic Memoranda*, respecting those diseases of the eye which are more frequently met with in practice. By JOHN FOOTE, Fellow of the Royal College of Surgeons in London, etc., etc. 12mo. Published by Samuel S. and William Wood, New-York. 1848.

"A delectable, pithie and righte profitable worke"—a genuine *multum in parvo*—containing a brief account of nearly every ophthalmic disease, and the most approved treatment, without theory, or speculation, or doubtful practice. The whole done up so snugly and compactly, that a man may now carry all the diseases of the eye, with their remedies also, in his vest pocket: such a vest pocket, we mean, of course, as a Frenchman carries his snuff in.

If, therefore, any doctor should feel it a privilege and comfort to be able always to carry about with him these numerous maladies, we recommend him to purchase "Foote's Ophthalmic Memoranda," at the store of F. W. Breed, bookseller, No. 188, Main st., Buffalo.

